# Comparison of corneal measurements in normal and keratoconus eyes using Anterior Segment Optical Coherence Tomography (AS-OCT) and Pentacam HR topographer

**DOI:** 10.1186/s12886-023-02946-w

**Published:** 2023-05-03

**Authors:** Omar M. Said, Mahmoud Kamal, Sara Tawfik, Ahmed Tamer Sayed Saif

**Affiliations:** Department of Ophthalmology, Faculty of Medicine, Fayoum University, Fayoum, Egypt

**Keywords:** Topographic Corneal Thickness, Keratoconus, Pentacam, AS-OCT

## Abstract

**Background:**

Keratoconus (KC) is easily recognized by its unique topographic pattern, but it can be difficult to distinguish subclinical form of the disease from the normal cornea. Optovue anterior segment optical coherence tomography (AS-OCT) helps diagnose KC.

**Aim of the work:**

To assess and the level of agreement of Keratometry-readings (K), Central Corneal Thickness (CCT) and Thinnest Corneal Thickness (TCT) measurements obtained by Optovue AS-OCT and Wavelight Oculyzer Pentacam HR in two groups: KC eyes and normal eyes.

**Patients and methods:**

This is a prospective clinical observational study. The study included 110 eyes divided into two groups. The study group included 62 eyes with topographic evidence of KC. The control group included 48 eyes of normal subjects with no topographic evidence of KC. All of the participants underwent full cycloplegic refraction, spectacle best-corrected distance visual acuity, comprehensive slit-lamp biomicroscopy and fundoscopy. All participants underwent corneal topography by Pentacam HR and AS-OCT.

**Results:**

There were highly significant differences between the studied groups as regarding BCVA, intraocular pressure and CCT measurements which were found to be lower among KC group compared to the control one. There were highly significant differences between the studied groups regarding TCT measurement detected by Pentacam HR and AS-OCT which was found to be lower among the keratoconus group compared to the control one (470.9, 455.7 versus 541.9 and 518.7 respectively).

**Conclusion:**

Both Scheimpflug-based imaging and AS-OCT provide comparable readings with a good agreement regarding corneal pachymetry in keratoconus group with accurate identification of KC eyes and healthy ones. However, there was a significant difference in K readings between both devices in Keratoconus and control group.

## Introduction

Keratoconus, the most common ectatic disorder, is characterized by the bilateral and progressive corneal thinning. Overall, focal thinning occurs in the inferior temporal region of the cornea, and identification of this characteristic corneal thinning pattern is a beneficial new method for diagnosing keratoconus [1].

The main treatment options includes corneal cross-linking, intracorneal rings implantation, photorefractive keratectomy, combined procedures (cross-linking plus) and keratoplasty [2–7].

While moderate to severe keratoconus is easily recognizable by the characteristic topographic pattern and classic clinical signs, it can be tough to differentiate subclinical forms of the disease from normal corneas, as patients usually present with normal visual acuity, stable topographic patterns and minimal or no clinical sign [8].

In these types, AS-OCT can produce reliable pachymetry maps that can identify Keratoconus and corneal thinning prior to laser refractive surgery [9].

Efforts have been made over the years to measure and quantify corneal thickness using several imaging systems. Accurate, precise, and reproducible measurements of corneal thickness are increasingly important in the decision-making process of refractive surgery, along with short-term and long-term postoperative evaluation. In addition, corneal thickness mapping is important for evaluation of ectatic corneal diseases like keratoconus [10].

Presently, imaging systems using slit-scanning technology such as Orbscan IIz, (Baush and Lomb, Rochester, NY, USA), a spectral-domain AS-OCT (RTVue-100, Optovue Inc, Fremont, CA, USA), and a high-resolution Scheimpflug rotating camera (WaveLight® Oculyzer™ II, Oculus Optikgeräte GmbH, Wetzlar, Germany) are now being used routinely in clinical settings. The reliability and reproducibility of these imaging techniques are significant for making clinical diagnoses, monitoring, and assessing treatment regimens [11, 12].

Recently, it has been shown that these anterior segment imaging techniques have been efficient in imaging the anterior segment parameters used to measure corneal pachymetry [13].

Optovue AS-OCT is a light-based imaging method that provides high-resolution images of the cross-sectional anterior segment of the eye. A non-contact detailed examination of the anterior segment up to 20 mm in diameter and the central 5 mm diameter area of ​​the pachymetry map can also be used to diagnose keratoconus [14].

The WaveLight® Oculyzer™ diagnostic device is based on proven Pentacam HR technology and enables non-contact measurement and analysis of the entire anterior segment. Measurements are taken from the anterior surface of the cornea to the posterior surface of the lens. The integrated rotatable Scheimpflug camera takes up to 50 images with real-time measurements at up to 25,000 measurement points [15].

### Patients and methods

Study design: this prospective clinical observational study was approved by the ethics committee of Fayoum Faculty of Medicine, Fayoum University, Egypt. The participants provided a written informed consent before the examination. The consent form was approved by the ethics committee. This study adhered to the tenets of the declaration of Helsinki. The protocol of this study was registered on www.clinicaltrials.gov (Registration number: NCT04462991).

Participants of the study (*n* = 56) were recruited from subjects seeking laser refractive surgery.

The study included 110 eyes divided into two groups;Group I; 48 eyes of 24 patients of normal subjects with no topographic evidence of KC.Group II; keratoconus patients. Sixty-two eyes of 32 patients with topographic evidence of KC ( stage I-III according to Amsler-Krumeich classification) [16].

Exclusion criteria were 1)Un-cooperative patients. 2)Patients with corneal opacity.3)Previous history of corneal surgery. 4) Corneal astigmatism of more than 4 diopters (for normal group). 4)Contact lens users.

All of the participants underwent full cycloplegic refraction, spectacle best corrected distance visual acuity, comprehensive slit lamp biomicroscopy and fundoscopy.

All participants underwent corneal topography by WaveLight® Oculyzer™II Pentacam HR and second by The Optovue AS-OCT as follows.

**Fig. 1:**
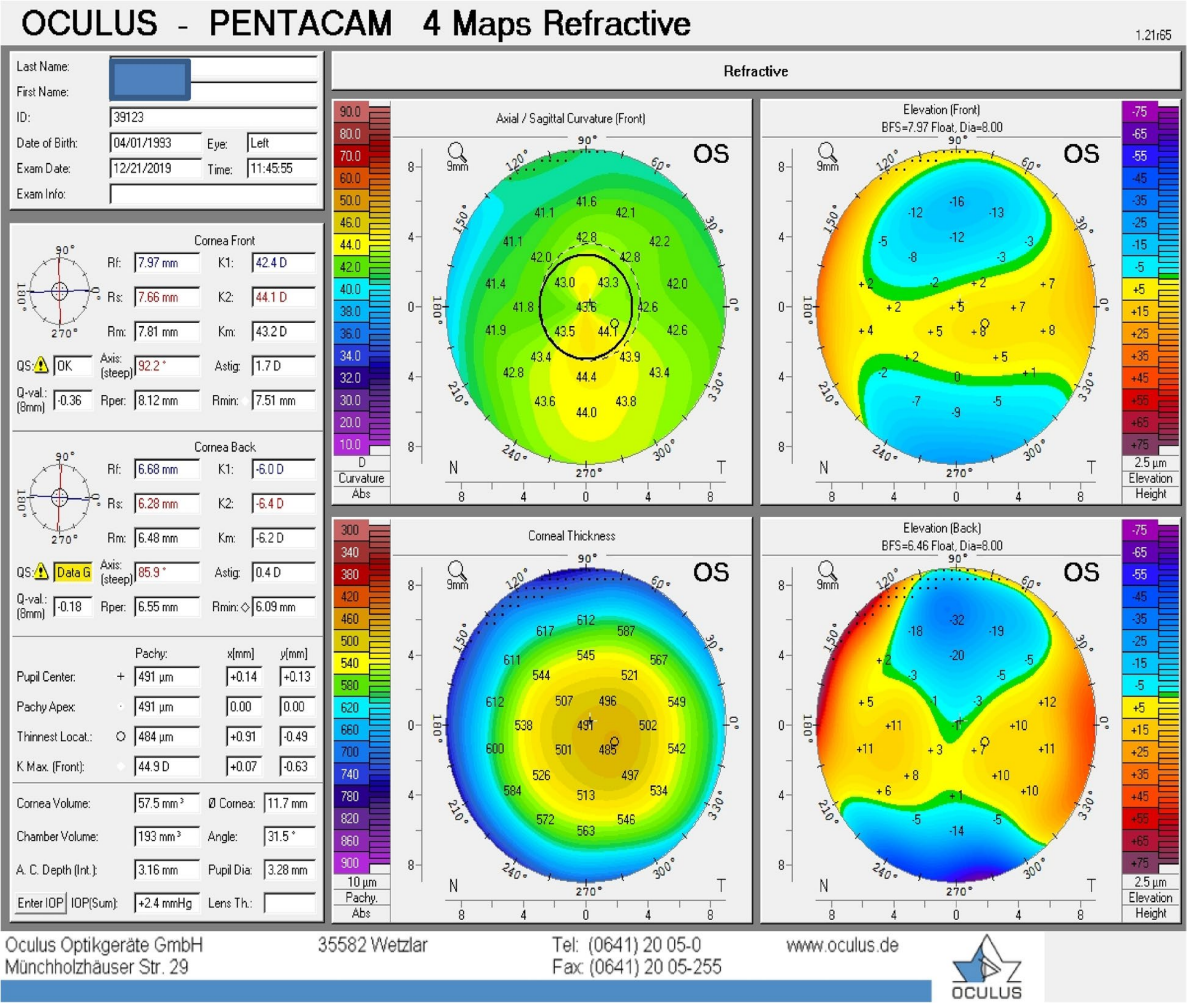
WaveLight® Oculyzer™ Diagnostic Device Printout of Keratoconus case

1. Corneal topography was done using the WaveLight® OculyzerTM Diagnostic Device which uses Scheimpflug imaging to provide simulated keratometry (in diopters), and corneal pachymetric maps (Fig. 1).
  Central Corneal Thickness (CCT),Thinnest Corneal Thickness (TCT),average keratometric power for anterior corneal surface and average keratometric power for posterior corneal surface.

### Corneal topography by AS-OCT system RTVue® 100 (Optovue, Fremont, CA, USA): (Fig.  2)

and Corneal pachymetry (TCT CCT), power (Anterior and Posterior keratometric measurements) and epithelial thickness maps were done using the Fourier-domain AS-OCT system RTVue® 100 (Optovue, Fremont, CA, USA) with a scan rate of 26 000 axial scans per second, the axial resolution of 5 μm, transverse resolution of 15 μm and an add-on lens (CAM-L mode: 6.0 − 2.0 mm).

**Fig. 2:**
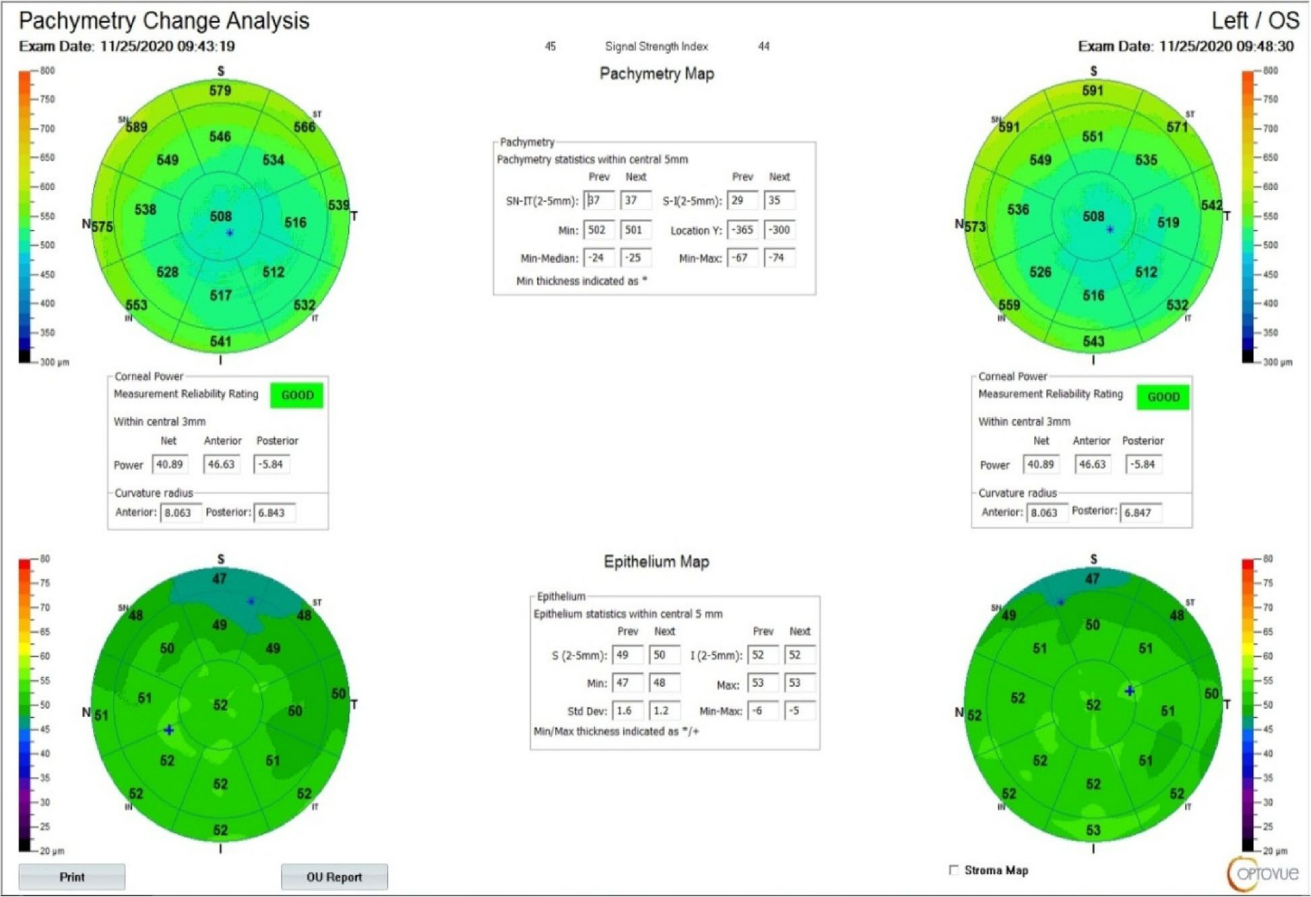
Fourier-domain AS-OCT system (Optovue, Fremont, CA, USA) printout

Patients were asked to fixate on the target light source and consecutive images were acquired with the patient’s forehead and chin stabilized by a headrest. Images were obtained in duplicate to confirm thickness measurement reproducibility. OCT has been shown to have excellent repeatability for total corneal thickness and power and epithelial thickness measurements [17].

A computer algorithm automatically maps total corneal thickness and corneal epithelial thickness across the central 6 mm of the corneal surface.

### Statistical analysis

All data were collected, tabulated, and statistically analyzed using SPSS version 19. Continuous Quantitative variables were expressed as the mean ± SD & (range), and categorical qualitative variables were expressed as absolute frequencies (number)& relative frequencies (percentage). Continuous data were checked for normality by using the Shapiro Wilk test. Independent samples Student's t-test was used to compare two groups of normally distributed data. Categorical data were compared using the Chi-square test (**χ**^**2**^test).

Pearson’s coefficient correlation test (r) was used to detect the closeness of association between two numeric variables.

All tests were two-sided. *P*-value < 0.05 was considered statistically significant (S), *p*-value < 0.001 was considered highly statistically significant (HS), and *p*-value ≥ 0.05 was considered statistically insignificant (NS).

The reliability of OCT was evaluated using Interclass Correlation Coefficient (ICC) and (95% confidence limits). ICC near 1 is considered a strong agreement between the readings.

## Results

The study included 110 eyes divided into two groups. The study group included 62 eyes with topographic evidence of KC (stage I-III according to Amsler-Krumeich classification). The control group included 48 eyes of normal subjects with no topographic evidence of KC.

Table 1 shows that there was a non-significant difference between the studied groups regarding age and sex.

**Table 1:** Comparison of demographic data among the studied groups

Variable	KC group		Control group		t-test	*P* value
	**(*n* = 62)**		**(*n* = 48)**			
**Age: (years)**						
Mean ± SD	25.9 ± 10.5		28.3 ± 5.1		-1.42	0.158
Range	Aug-56		20—38			(NS)
	**No**	**%**	**No**	**%**	**χ**^**2**^	**P**
**Sex:**						
Female	33	53.2	34	70.8	3.523	0.06
Male	29	46.8	14	29.2		(NS)

*NS* Non-significant difference (*p* > 0.05)

There were highly significant differences between the studied groups regarding intra-ocular pressure which was found to be lower among the keratoconus group compared to the control one (12.3 versus 14.8 respectively) (Table 2).

**Table 2:** Comparison of intra-ocular pressure among the studied groups

Variable	KC group	Control group	t-test^a^	*P* value
	**(*n* = 62)**	**(*n* = 48)**		
**IOP:**				
Mean ± SD	12.3 ± 2.8	14.8 ± 1.8	**-5.386**	** < 0.001**
Range	19-Jun	19-Dec		**(HS)**

*HS* Highly significant difference (*p* < 0.001)

^a^t-test between KC and control groups

Table 3 illustrated that there were highly significant differences between the studied groups regarding CCT measurements detected by both Pentacam and AS-OCT which were found to be lower among the keratoconus group compared to the control one (485.8, 475.1 versus 543.9 and 529.6 respectively). By comparing CCT measurements among the control group by both techniques, it was found that there was a significant difference between them;however it was non-significant among the Keratoconus group.

**Table 3:** Comparison of CCT by PENTACAM and by OCT among the studied groups

**Variable**	**KC group**	**Control group**	**t-test**^a^	***P* value**
	**(*n* = 62)**	**(*n* = 48)**		
**CCT by PENTACAM:**				
Mean ± SD				
Range	485.8 ± 40.7	543.9 ± 28.1	**-8.392**	** < 0.001**
	393—586	496—616		**(HS)**
**CCT by**				
**OCT:**				
Mean ± SD	475.1 ± 39.5	529.6 ± 30.8	**-7.878**	** < 0.001**
Range	388—571	481—597		**(HS)**
**P**^b^**:**	0.139 (NS)	**0.008 (S)**		

*HS* Highly significant difference (*p* < 0.001), *NS* Non-significant (*p* > 0.05)

^a^t-test between KC and control groups

^b^t-test between PENTACAM and OCT

Table 4 illustrated that there were highly significant differences between the studied groups regarding TCT measurement detected by PENTACAM and OCT which was found to be lower among the keratoconus group than the control one (470.9, 455.7 versus 541.9 and 518.7 respectively). By comparing TCT measurements among the control group by both techniques, it was found that there was a highly significant difference between them. TCT measurements by PENTACAM were significantly higher than that detected by OCT (541.9 versus 518.7 respectively).

**Table 4:** Comparison of TCT by PENTACAM And OCT among the studied groups

**Variable**	**KC group**	**Control group**	**t-test**^a^	***P* value**
	**(*n* = 62)**	**(*n* = 48)**		
**TCT by PENTACAM:**				
Mean ± SD				
Range	470.9 ± 44.2	541.9 ± 29.4	**-9.584**	** < 0.001**
	329—567	489—612		**(HS)**
**TCT by**				
**OCT:**				
Mean ± SD	455.7 ± 46.6	518.7 ± 26.2	**-8.377**	** < 0.001**
Range	252—558	475—584		**(HS)**
**P**^b^**:**	0.06 (NS)	** < 0.001 (HS)**		

*HS* Highly significant difference (*p* < 0.001), *S* Significant difference (*p* < 0.05)

^a^t-test between KC and control groups

^b^t-test between PENTACAM and OCT

**Fig. 3:**
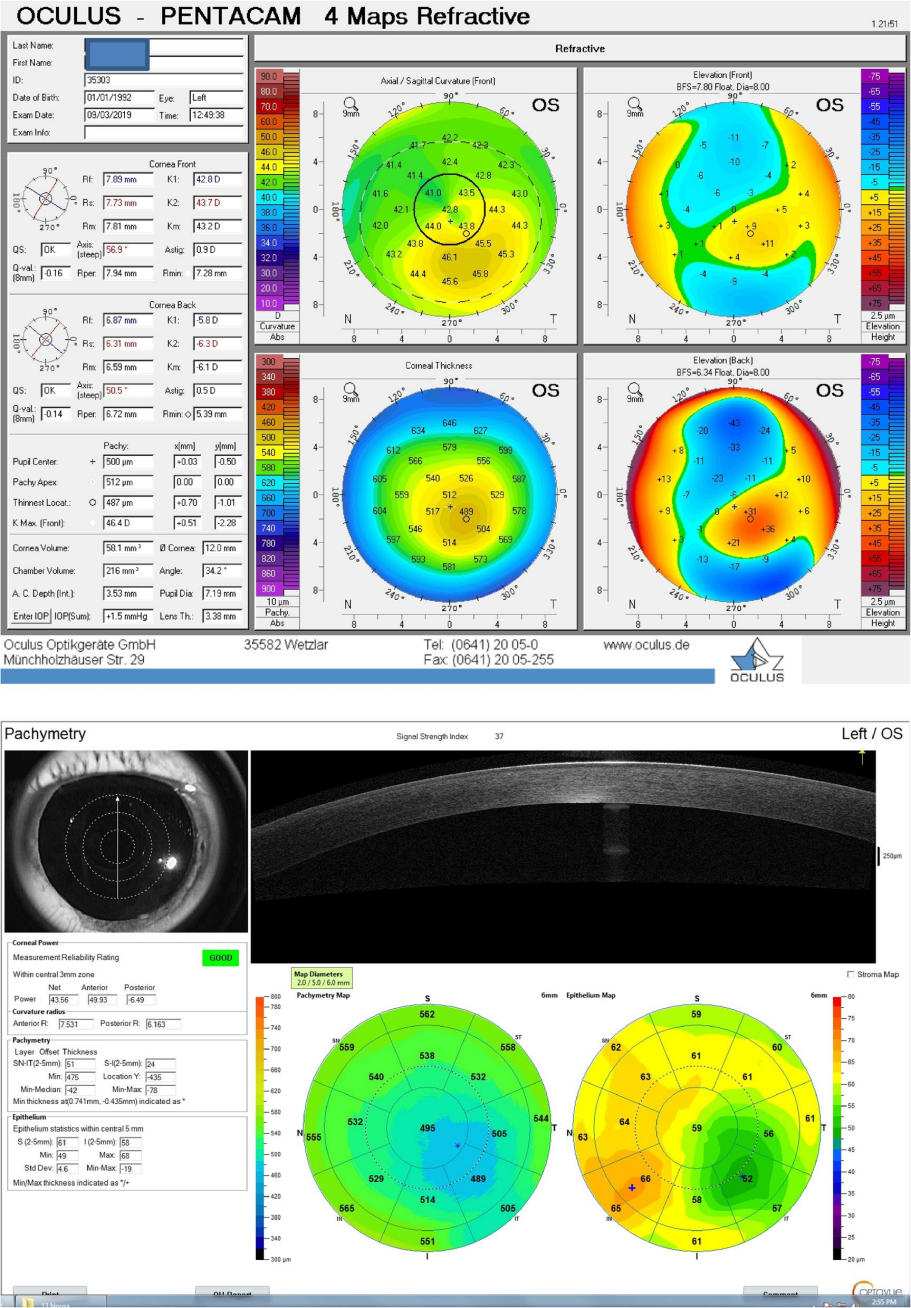
Corneal Pachymetric and keratometric measurements as obtained by WaveLight® Oculyzer™ II (upper) and by Fourier-domain AS-OCT system RTVue® 100 (lower) Diagnostic Device

**Fig. 4:**
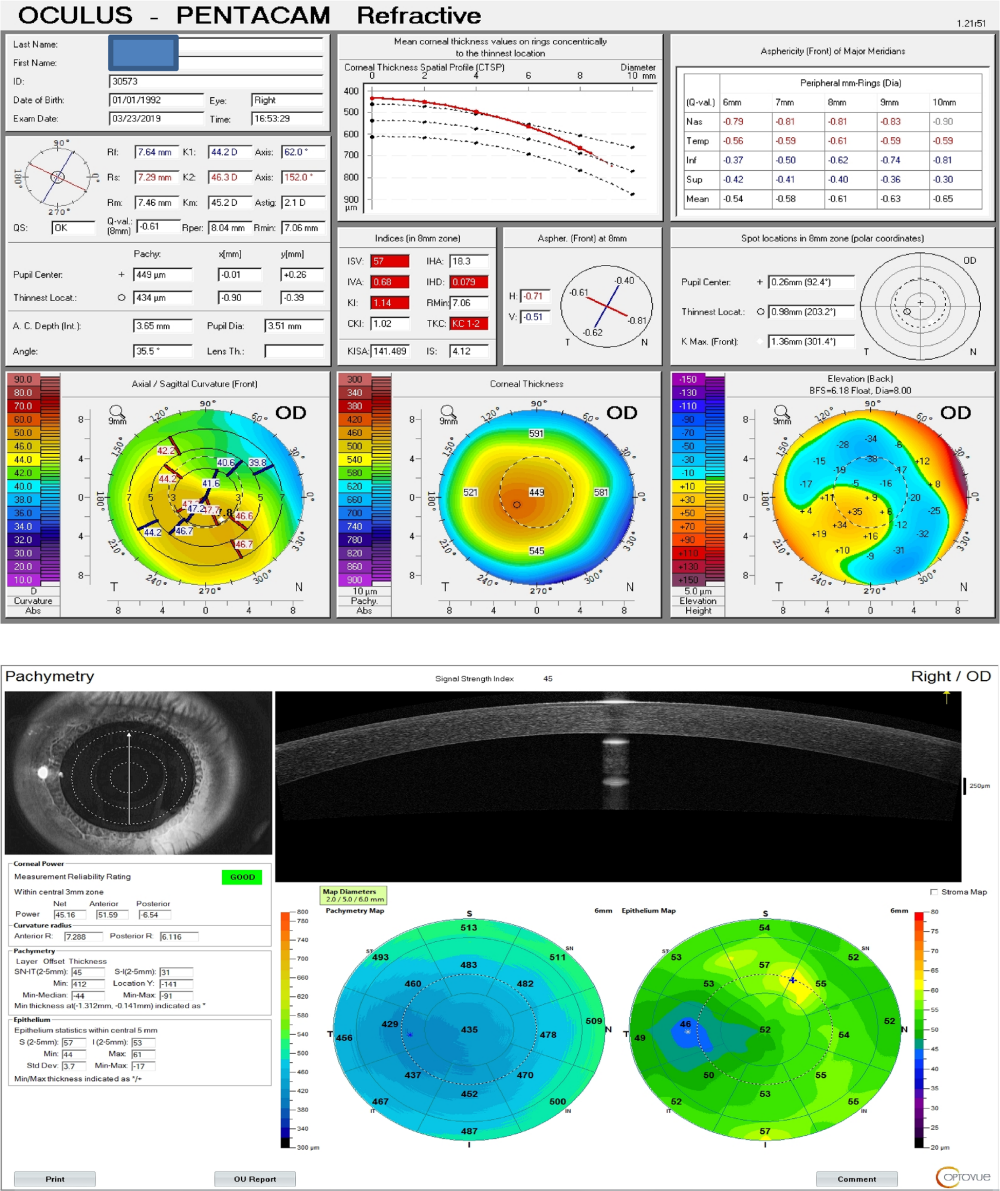
Corneal Pachymetric and keratometric measurements as obtained by WaveLight® Oculyzer™ II (upper) and by Fourier-domain AS-OCT system RTVue® 100 (lower) Diagnostic Device

Although the TCT measurements difference was found to be non-significant among KC group by both techniques, they were found to be lower by AS-OCT.(Figs. 3 &4).

Table 5 shows that there was a significant difference between the studied groups as regarding anterior mean K measurements detected by PENTACAM which was found to be higher among the keratoconus group compared to the control one (47.1 versus 43.6 respectively). And there was a highly significant difference between the studied groups regarding anterior mean K measurements detected by OCT which was found to be higher among the keratoconus group compared to the control one (52.3 versus 47.8 respectively).

**Table 5:** Comparison of K anterior values by PENTACAM and OCT among the studied groups

Variable	KC group	Control group	t-test^a^	*P* value
	**(*n* = 62)**	**(*n* = 48)**		
**K anterior by PENTACAM:**				
Mean ± SD				
Range	47.1 ± 3.5	43.6 ± 1.4	**6.408**	**0.001**
	42.8—57	41.1 – 47.2		**(S)**
**K anterior by OCT:**				
Mean ± SD				
Range	52.3 ± 4.2	47.8 ± 2.4	**6.511**	** < 0.001**
	45.6 – 64.5	41.8 – 53.1		**(HS)**
**P**^**b**^**:**	** < 0.001 (HS)**	** < 0.001 (HS)**		

*HS* Highly significant difference (*p* < 0.001), *S* Significant difference (*p* < 0.05)

^a^t-test between KC and control groups

^b^t-test between PENTACAM and OCT

By comparing K anterior measurements among the KC group by both techniques (Figs. 3 &4), it was found that K anterior by OCT was significantly higher than that detected by PENTACAM (52.3 versus 47.1 respectively). The same significance was found in the control group.

Table 6 shows that there was a significant difference between the studied groups as regarding K posterior measurements detected by PENTACAM and OCT which was found to be higher among the keratoconus group compared to the control one (-6.88, -7.09 versus -6.25 and -5.90 respectively). By comparing K posterior measurements among the KC group by both techniques, it was found that there was a non-significant difference between them.

**Table 6:** Comparison of K posterior values by PENTACAM and OCT among the studied groups

Variable	KC group	Control group	t-test^a^	*P* value
	**(*n* = 62)**	**(*n* = 48)**		
**K posterior by PENTACAM:**				
Mean ± SD				
Range	-6.88 ± 0.83	-6.25 ± 0.20	**-5.129**	**0.001**
	(-8.9) – (-4.4)	(-6.7) – (-5.9)		**(S)**
**K posterior by OCT:**				
Mean ± SD				
Range	-7.09 ± 1.1	-5.90 ± 1.81	**-4.329**	**0.001**
	(-9.8) – (-4.9)	(-6.6) – (6.3)		**(S)**
**P**^**b**^**:**	0.235 (NS)	0.187 (NS)		

*S* Significant difference (*p* < 0.05)

^a^t-test between KC and control group

^b^t-test between PENTACAM and OCT

Table 7 shows a high degree of agreement ( good repeatability)between the two readings of central epithelium, CCT, TCT, K-anterior and K-posterior obtained by AS-OCT with interclass correlation coefficient of 0.890, 0.992, 0.986, 0.996 and 0.980 respectively.

**Table 7:** Interclass Correlation Coefficient (ICC) of different parameters

Variable	ICC (96%CI)*
**Cenral epithelium:** A1, A2	**0.890(0.76–0.94)**
**2003CCT:** A1, A2	**0.992(0.98–0.99)**
**TCT:** A1, A2	**0.986(0.97–0.99)**
**K-anterior:** A1, A2	**0.996(0.992–0.998)**
**K-posterior:** A1, A2	**0.980(0.96–0.98)**

*Two-way random model, absolute agreement

A1: first measurement by the examiner, A2: second measurement by the examiner

There were highly significant differences between the studied groups regarding central epithelium which was found to be lower among the keratoconus group compared to the control one (49.1 versus 53.8 respectively).

**Fig. 5:**
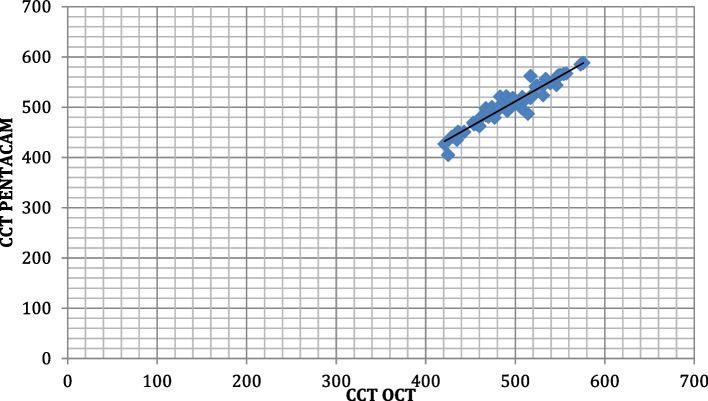
Correlation between CCT measurements by both techniques among the studied participants

**Fig. 6:**
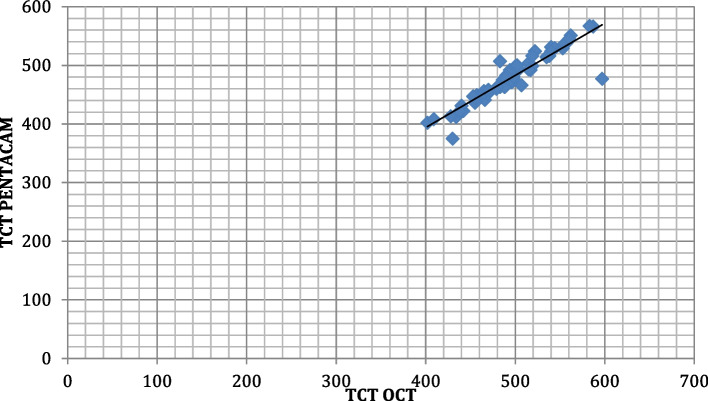
Correlation between TCT measurements by both techniques among the studied participants

The correlation between CCT measurements obtained by OCT and PENTACAM among the studied group shows that there was a strong positive highly significant correlation between CCT when measured by both PENTACAM and OCT. (r 0.996, *p* < 0.001*)(Fig. 5). The correlation between TCT measurements obtained by OCT and PENTACAM among the studied group shows that there was a strong positive highly significant correlation between TCT when measured by both PENTACAM and OCT (r0.929, *p* < 0.001*)(Fig. 6).

**Fig. 7:**
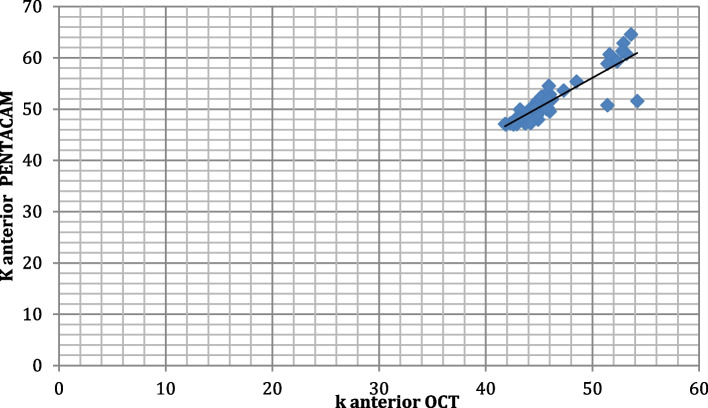
Correlation between K anterior measurements by both techniques among the studied participants

The correlation between K anterior measurements obtained by OCT and PENTACAM among the studied group shows that there was a strong positive highly significant correlation between k anterior when measured by both PENTACAM and OCT (r0.838, < 0.001*)(Fig. 7).

The correlation between K posterior measurements obtained by OCT and PENTACAM among the studied group: shows that there was a strong positive highly significant correlation between k posterior when measured by both PENTACAM and OCT (0.596, < 0.001*).

## Discussion

This work examined the corneal parameters in a group of KC eyes and healthy control eyes utilizing two different methods: Pentacam and AS-OCT. Our findings suggest that most of the keratometric values cannot be used interchangeably between two devices with significant inter-method variability.

In a recent study by Gim et al., Scheimpflug imaging was compared to AS-OCT measurements in normal eyes. It was found that there are statistically significant differences in keratometric measurements not acceptable for interchangeable use. Which is in agreement with our study results. However, the study did not attempt to define interchangeability in KC eyes [18]. In contrast to our study, Herber et al. also assessed a novel swept source OCT machine and compared the readings to those obtained from Pentacam measurements, and the Galilei G6 (dual Scheimpflug-Placido system). The results showed that all three machines had comparable readings. Yet, all studied eyes were normal ones [19].

Li et al., studied (CCT) and (TCT) in KC eyes using both Pentacam and AS-OCT measurements. Although both machines demonstrated good repeatability, there was poor agreement between different groups [20]. This contrasts with our work where a good agreement for CCT & TCT between both machine readings was demonstrated in KC patients. However, a high statically significant difference regarding both parameters in the healthy control group was recorded. The late finding is supported by a novel study conducted on 206 healthy eyes comparing corneal thickness obtained by Pentacam, Specular microscopy, IOL Master and AS-OCT found that CCT measurements of Pentacam were statistically higher than that of AS-OCT which is in agreement with our study results [21].

It is to be noted that a lot of variables can affect measurement of the thinnest location using either technique. A study from Japan Fujimoto et al., recently demonstrated that both CCT and TCT measurements were markedly increased in patients with severe dry eye disease when measured using pentacam or AS-OCT [22]. Furthermore, the location of the thinnest location as detected by either machine markedly deviated. This demonstrates that corneal surface dryness can be a great confounding factor when it comes to measuring corneal thickness using pentacam or AS-OCT. The presence and degree of corneal oedema can also be a contributing variable when it comes to corneal thickness assessment using different modalities [22]. In the latter case, ultrasound pachymetry should be avoided, and both Scheimpflug-based techniques or AS-OCT were demonstrated to be in agreement.[23]This was also confirmed by another study Wongchaisuwat et al., that demonstrated good agreement between methods in measuring CCT up to 650 µm; however, in corneal thicknesses above 650 µm, Pentacam measurements tended to be overestimated [24]. Another demonstrated factor that may contribute to inconsistent measurements is diabetes mellitus. A study demonstrated that when measuring the corneal thickness in eyes of diabetic patients, the Pentacam machine overestimated the CCT. This was attributed to the diabetic condition altering the tear film status and acting as a cause of severe dry eye [25]. In those cases, AS-OCT may be a better option. This supports the findings of Maloca and colleagues that demonstrated the superiority of AS-OCT in reproducible CCT measurements [26].

Only a few studies attempted to assess variability between Scheimpflug-based imaging and AS-OCT in KC eyes. A study on this subject showed significant differences in posterior corneal surface and corneal thickness measurements between swept-source OCT and combined Placido–Scheimpflug imaging in eyes with Keratoconus [27]. Both are in agreement with our study regarding keratometeric values.

A recent study that looked for agreement between AS-OCT and Sirius devices was conducted on 44 KC patients and found a significant difference in Keratometry values which is agreed with our study in which we found a highly significant difference in K anterior readings between OCT and Pentacam with higher readings in OCT [28]. On the other hand, a study conducted on 30 patients in China comparing the same devices in KC patients found a significant difference in K posterior as well [27].

Another study Gjerdrum et al., on two different OCT machines, showed that simulated K readings were highly variable in both machines when compared to Pentacam measurements, and suggested better reliability of Scheimpflug-based imaging when it comes to keratometric parameters [29]. On the other hand, Zhang and colleagues demonstrated no difference in clinical outcome when assessing keratometric readings using both machines [30].

Regarding central epithelial thickness (CET), recent studies showed that corneal epithelial thickness mapping by OCT could be useful to detect an incipient corneal ectasia in clinically and topographically normal eyes [31, 32]. In this context, Haque, Simpson, and Jones measured CET in normal and keratoconic eyes using AS-OCT demonstrated that central keratoconic epithelium was thinner than the normal cornea, this is in agreement with our study [33].

Moreover, W. Zhou and Stojanovic revealed that the epithelium and stroma in keratoconic eyes were thinner inferotemporally and thicker supranasally compared with control eyes with more distinct patterns in epithelium than in the stroma [34]. An up to date study, Jhanji et al., revealed that AS-OCT images could accurately characterize the epithelial and corneal thickness changes at different stages of the KC progression [35].

Lastly, measurement of corneal topographic and thickness parameters is crucial in the clinical management of KC, since major decisions are often based on the measurements (e.g. observation, cross-linking, or corneal grafting) [36]. Therefore, strict quality control on ophthalmological imaging is important to obtain the highest quality, reproducible readings [37]. This can be achieved through operator training, good communication between the treating ophthalmologist and the imaging personnel, a detailed description of each effect, and auditing of reporting errors [37].

The strength of our study is that it is the only study to assess both K-readings and corneal thickness parameters by Pentacam and AS-OCT in two groups: KC eyes and normal eyes. The results demonstrate moderate inter-device changeability between both devices.

Limitations to our study include the relatively small sample size. Future studies with larger sample sizes could corroborate our findings. We also did not include different grades of KC, or eyes with different prior treatments; both parameters would be expected to induce measurement variability. Finally, long-term prospective studies with follow up measurements are warranted.

## Conclusion

Both Scheimpflug-based imaging and AS-OCT provide comparable readings with a good agreement regarding corneal pachymetry with accurate identification of KC eyes and healthy ones. However, due to the significant difference in K readings between both devices, we do not recommend using the two devices inter-changeably. Future prospective studies with larger sample sizes are warranted to document any inter-device differences, especially among different grades and different treatment modalities of KC.

## Data Availability

Any additional Data are available upon request to corresponding author.
